# Adaptive, Bayesian Experimental Design to Efficiently Determine the Critical Micelle Concentration of a Surfactant

**DOI:** 10.1021/acs.langmuir.5c06514

**Published:** 2026-04-07

**Authors:** Makaila Hammond, Qia Ke, Aryan Deshwal, Cory M. Simon, Alison Bain

**Affiliations:** † Department of Chemistry, 2694 Oregon State University, Corvallis, Oregon 97331-4501, United States; ‡ School of Chemical, Biological, and Environmental Engineering, Oregon State University, Corvallis, Oregon 97331-4501, United States; ¶ Department of Computer Science and Engineering, University of Minnesota, Minneapolis, Minnesota 55455, United States

## Abstract

Surfactants are widely used for industrial applications, yet more environmentally friendly surfactants with enhanced properties are demanded. A key thermodynamic property governing the behavior of a surfactant in an aqueous solution is its critical micelle concentration (CMC). Below the CMC, increasing the surfactant concentration reduces the surface tension of the solution; above the CMC, the water–air interface becomes saturated with adsorbed surfactant, leading excess surfactant to self-assemble into micelles and the surface tension to plateau. Many physicochemical properties of a surfactant solution exhibit sharp changes at the CMC. The conventional experimental protocol to determine the CMC of a surfactant is labor-intensive and time-consuming: (1) prepare many surfactant solutions spanning a wide concentration range and then (2) measure the surface tension of each solution. Herein, we adopt Bayesian experimental design (BED) to determine the CMC of a surfactant more efficiently—even without prior knowledge of its order of magnitude. BED follows an experiment-model-design feedback loop: (1) prepare a surfactant solution and measure its surface tension; (2) use all surface tension data thus far to obtain a posterior distribution over thermodynamic models of the surface tension isotherm of the surfactant; and (3) pick the surfactant concentration for the next experiment to maximize expected information gain about the CMC. We show that BED efficiently gathers information about the CMC using two surfactants (octyl-β-d-thioglucopyranoside and Triton X-100) as test cases. Broadly, BED can reduce the time, effort, cost, and chemical waste to determine the CMC of surfactants and drive an autonomous laboratory for surfactant discovery and characterization.

## Graphical abstract

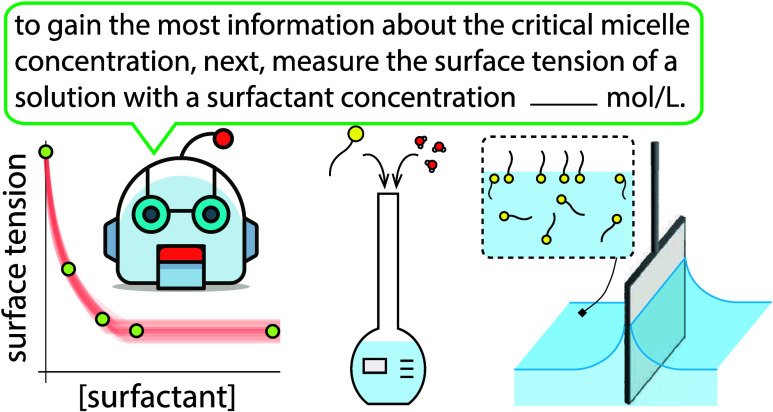

## Introduction

A *surfactant* (SURFace ACTing AgeNT)^1^ is an organic molecule composed of a hydrophobic subunit connected to a hydrophilic subunit. This dual structure imparts surfactants with the ability to strongly adsorb on a surface or at an interface in a directional manner; reduce the surface (interfacial) tension of a water–gas (water–oil) interface; and self-assemble into micelles in the bulk of an aqueous solution. Thanks to these properties, surfactants have a multitude of applications functioning as wetting agents, emulsifiers, dispersants, solubilizers, mobilizers, foaming agents, and lubricants. For example, surfactants are active ingredients or additives in detergents, personal hygiene products (e.g., shampoo, facial cleansers, soap), pharmaceutical formulations, food products, coatings, paints and inks, agrochemicals, injectants for enhanced oil recovery, and formulations for the remediation of water and soil contamination.^2^ While most surfactants for industrial applications are synthesized from petrochemical or oleochemical feedstocks, many [bio]­surfactants are naturally occurring.^3^

The *surface tension isotherm* of a surfactant in an aqueous solution examines the surface tension of the solution as a function of bulk surfactant concentration at constant temperature [and pressure].^4^ Surfactant molecules adsorb at an aqueous interface, oriented for the hydrophilic portion to interact with water while the hydrophobic portion interacts with air or oil.^5^ These adsorbed surfactant molecules disrupt the hydrogen bonding network of water molecules, reducing the tension at the interface. See refs. 6,7 for a refresher on surface tension. At low surfactant concentrations, increasing the surfactant concentration decreases the surface tension of the interface. However, once the *critical micelle concentration* (CMC) is reached, the interface becomes saturated with a monolayer of adsorbed surfactant molecules. Consequently, additional surfactant added to the solution self-assembles into micelles in the bulk solution.^5,8^ Micelles are (often, spherical) microstructures of aggregated surfactant molecules where the hydrophobic units lie in the interior core to avoid interacting with water, while the hydrophilic portions interact with the water and form a protective outer shell. As an example, Figure 1 illustrates the surface tension isotherm and CMC of the surfactant octyl-β-D-thioglucopyranoside (OTG).

**Figure 1:**
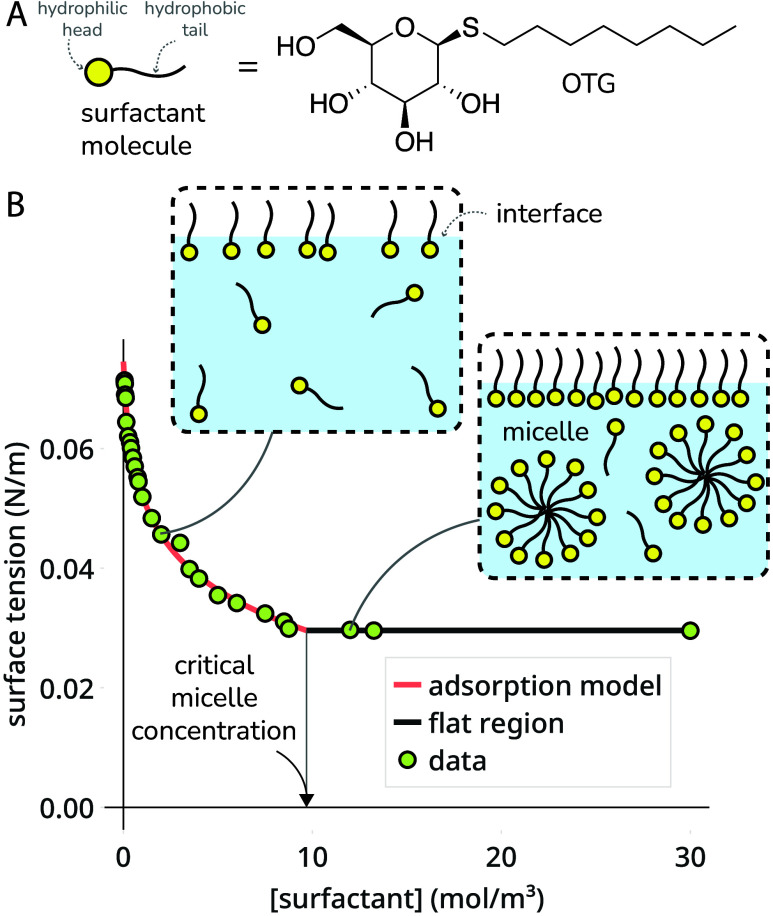
A surfactant, its surface tension isotherm, and its critical micelle concentration. (A) The surfactant molecule is octyl-β-d-thioglucopyranoside (OTG). (B) The surface tension isotherm of OTG in an aqueous solution (measured by us) at room temperature and its critical micelle concentration (CMC).

The CMC of a surfactant is an important thermodynamic property. Emergent from the chemical structure of the surfactant, the CMC dictates (a) the minimum concentration of surfactant that yields micelles, (b) the maximum concentration of surfactant that enables modulation of surface tension, and (c) a critical concentration at which many other bulk solution properties of interest, such as electrical conductivity and turbidity, sharply change.^9^ The formation of micelles is particularly key for surfactants to function as detergents and solubilizers as micelles can encapsulate oils, dirt, drugs, and hydrophobic water contaminants. Additionally, the CMC may be indicative of surfactant concentrations associated with toxicity,^10^ antimicrobial properties,^11^ and phenomena in synthetic cells.^12^ From surfactant-to-surfactant, CMCs vary across orders of magnitude. Additionally, the CMC depends on temperature and pressure (weakly) as well as the concentrations of ions in the solution.^5^

Experimentally determining the CMC of a surfactant in an aqueous solution is a routine task. First, given a new surfactant, we wish to know its CMC—a key property. Indeed, the design and discovery of new/alternative surfactants is driven by the need for more environmentally friendly and performant surfactants for existing and emerging applications.^13–15^ Second, we may wish to know the CMC of an existing surfactant under new conditions and contexts (e.g., temperatures, pHs, or concentrations of additional solutes).

The tensiometry approach to determine the CMC of a surfactant in an aqueous solution comprises two stages. First, in the laboratory, we collect data characterizing the surface tension isotherm of the surfactant. Specifically, we prepare a large number of solutions spanning [possibly, without prior information] orders of magnitude in surfactant concentration and then measure the surface tension of each solution via the Wilhelmy plate, pendant drop, or Du Noüy ring method.^16^ As an alternative to tensiometry, the CMC of a surfactant can be identified by measuring other solution properties that sharply change at the CMC—such as electrical conductivity, fluorescence, and light scattering—as a function of surfactant concentration.^9,17,18^ Second, computationally, we identify the CMC from the data by invoking some mathematical definition of the CMC or fitting a surface tension isotherm model.^9,19^

Herein, our aim is to reduce the number of experiments commonly expended to determine the CMC of a surfactant. To do so, we adopt Bayesian experimental design (BED)^20,21^ to decide on a sequence of surfactant concentrations in aqueous solutions for surface tension measurements to gather information about the CMC of a surfactant. Notably, BED has been applied to maximize information gain for several applications in the chemical sciences.^22–24^ At a high-level, BED constitutes iterating a feedback loop between (1) preparing a surfactant solution and measuring its surface tension; (2) updating a probabilistic model of the surface tension isotherm of the surfactant; and (3) deciding the surfactant concentration for the next experiment. Each decision is

- *adaptive*, i.e., based on all surface tension isotherm data collected thus far in the experimental sequence. In contrast, a static design sets the surfactant concentrations at the beginning of the experimental sequence.
- *judicious*, via being grounded by (a) a thermodynamic theory of surfactant adsorption at the interface and consequent modulation of the surface tension; (b) the [albeit, incomplete] surface tension isotherm data obtained thus far; and (c) decision-making principles based on information theory.
- *automatic*, as, after the human designs the BED algorithm, the computer picks the surfactant concentration for the next experiment.
- made under a Bayesian framework^25,26^ for (a) quantifying uncertainty about the surface tension isotherm of the surfactant at each stage of the experimental sequence and (b) incorporating prior information about the CMC of the surfactant (e.g., the range in which the CMC lies, based on the similarity of the chemical structure of the surfactant with other surfactants whose CMCs are known) for further experiment efficiency.

We devise our BED algorithm to estimate the CMC of a surfactant with high credibility using the fewest experiments—reducing the time, effort, cost, and chemical waste commonly expended to do so with static experimental designs. BED constitutes “active learning”, whereby we design each experiment to learn the most about the CMC of the surfactant. We demonstrate and explain how BED designs an efficient sequence of experiments for determining the CMC of two surfactants: (1) OTG and (2) Triton X-100, even when little prior information about (for Triton X-100, the order of magnitude) the CMC was provided. Compared to outcomes of static, [log]­uniform experimental designs simulated by a posterior-derived oracle, we find that BED reduces the number of experiments required to determine the CMC by up to a factor of 2.

Very recently, Dankloff et al.^27^ also reported using BED to orchestrate an autonomous robotic pendant drop module to characterize the surface tension isotherm of a surfactant in an aqueous solution. To highlight differences with our work, Dankloff et al.^27^ (1) initialized their experimental sequence with four times as many surface tension measurements as us—starting with a stock surfactant solution above the estimated CMC and sequentially diluting each solution by 2× to reach 1/128 the concentration of the stock solution—and (2) did not compare the experiment efficiency of BED with a static experimental design. Our paper serves more as a guidebook on BED for efficiently determining the CMC of surfactants by didactically expanding on the methodology.

## Methods

Our BED algorithm constitutes the feedback loop shown in Figure 2: (1; experiment) prepare a surfactant solution of a given concentration and measure its surface tension; (2; model) use the new data point −a (surfactant concentration, surface tension) pair– to update a probabilistic, parametric, thermodynamic model of the surface tension isotherm of the surfactant; then (3; design) algorithmically pick the surfactant concentration for the next experiment. Specifically, taking a Bayesian approach, we maintain a posterior probability distribution over the parameters of the surface tension isotherm model—including the CMC. This posterior distribution expresses our belief, grounded in data, about the surface tension isotherm, including the value of the CMC. As our decision-making principle, we pick the surfactant concentration for the next experiment that maximizes the information we expect to gain about the CMC. Sensible criteria for terminating the loop include obtaining a sufficiently narrow credible interval for the CMC or exhausting a budget of experiments.

**Figure 2:**
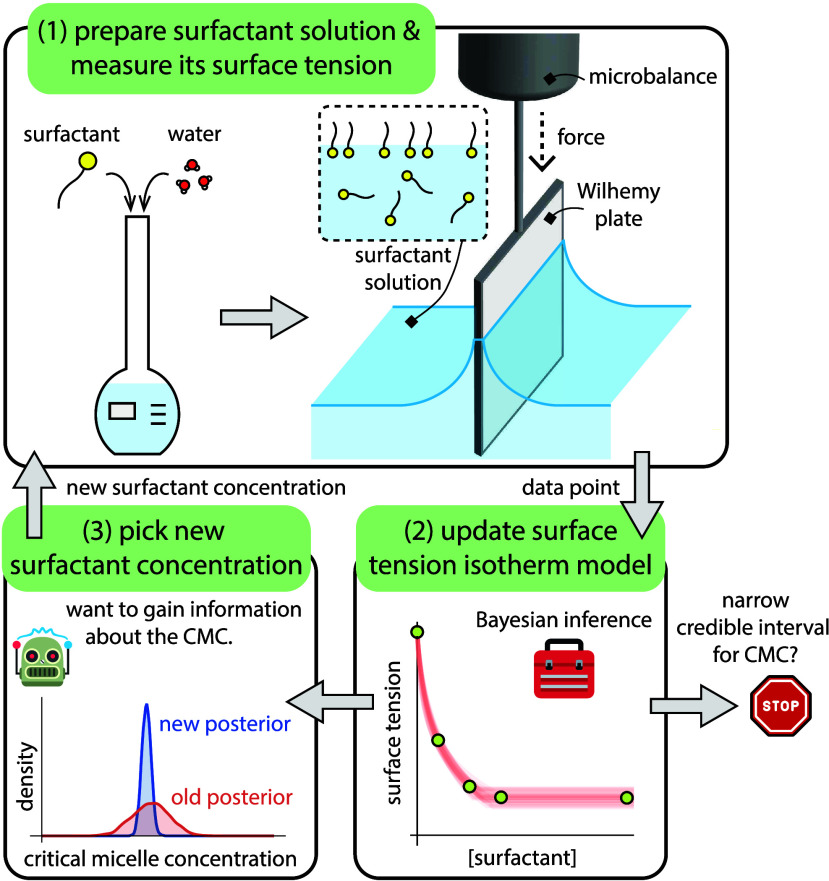
Adaptive Bayesian experimental design (BED) to experimentally and efficiently determine the critical micelle concentration (CMC) of a surfactant in an aqueous solution. Our BED algorithm iterates a feedback loop: (1) prepare a surfactant solution and measure its surface tension via the Wilhelmy plate method; (2) use the data to update a probabilistic, parametric thermodynamic model of the surface tension isotherm; then (3) judiciously pick the surfactant concentration for the next experiment to maximize expected gain of information about the CMC. A stopping criterion to terminate the loop is when the credible interval for the CMC is sufficiently narrow.

### Experimental Methods

#### Chemicals

We used the surfactant Triton X-100 (Sigma-Aldrich, laboratory grade) and the surfactant *n*-Octyl-β-d-thioglucopyranoside (OTG, Thermo Fisher Scientific, electrophoresis grade, ≥98% pure) without any further purification.

#### Preparing Surfactant Solutions

To prepare a concentrated stock solution of surfactant, we weighed surfactant on an analytical balance (with precision of ±0.0001 g) and diluted it in a volumetric flask with ultrapure water (electrical resistivity of 18 MΩ·cm). To prepare solutions with lower surfactant concentrations, we diluted this concentrated stock solution.

#### Force Tensiometry

To measure the surface tension of a surfactant solution at ambient temperature (25 °C) and pressure, we used a DataPhysics Instruments DCAT 8T Force Tensiometer with a Wilhelmy plate PT 11 probe made of platinum. The Wilhelmy plate method determines surface tension from the downward force measured on the wetted platinum plate, dipped into the solution with orientation normal to the interface.^28^ Preceding each measurement, we cleaned the probe with ultrapure water and dried it to red-hot via exposure to a butane flame. We measured the surface tension of each solution in triplicate and reported the average value.

#### Surface Tension Isotherm Data

A sequence of *N* experiments for a single surfactant generates a data set consisting of *N* data points:

1 $$D={\left\{\left(c_{i},\gamma_{i,\mathrm{obs}}\right)\right\}}_{i=1}^{N}$$

where *c*_*i*_ [mol/m^3^] is the concentration of the surfactant in solution *i* and γ_*i*,obs_ [N/m] is its measured surface tension.

### Computational Methods

#### Parametric Thermodynamic Model of the Surface Tension Isotherm

Our BED algorithm relies upon a parametric model of the surface tension isotherm of the surfactant, γ­(*c*; θ) with γ [N/m] being the surface tension of the solution, *c* [mol/m^3^] being the bulk surfactant concentration, and $\theta \in R^{l}$ being a parameter vector that includes the CMC c* [mol/m^3^] as a component; i.e., our BED algorithm assumes the true surface tension isotherm of the surfactant belongs to a family of functions γ­(*c*; θ) parametrized by θ. The model γ­(*c*; θ) could be (a) empirical or (b) grounded in thermodynamic theory, which introduces an inductive bias that could make inference of the CMC more data-efficient.

For our case studies, we assume the surface tension isotherm of the surfactant is (1) described by the Langmuir-Szyszkowski equation^29^ when the surfactant concentration is below the CMC and (2) flat when the surfactant concentration is at or higher than the CMC. While originally thought to be an empirical relationship,^30^ the Langmuir-Szyszkowski equation is grounded in thermodynamic theory.^4,31^ Particularly, it is obtained from the Gibbs adsorption isotherm equation^4^ provided surfactant adsorption at the interface follows the Langmuir model.^32^ Specifically, our surface tension isotherm model is

$$\gamma \left(c;\theta ≔\left[\gamma_{0},a,k,c^{*}\right]\right)=\{\begin{array}{cc} \gamma_{0}-a\log\left(1+\mathrm{kc}\right) & c<c^{*}\\ \gamma_{0}-a\log\left(1+kc^{*}\right) & c\geq c^{*} \end{array}$$ 2

which contains l=4 parameters in the vector θ: γ_0_ [N/m], the surface tension of the pure solvent (water); *a* [N/m], related to the cross-sectional area occupied by a surfactant molecule at the interface; *k* [m^3^/mol], the Langmuir equilibrium constant describing the affinity of the surfactant for the interface; and c* [mol/m^3^], the CMC of the surfactant. The parameters *a*, *k*, and c* differ from surfactant-to-surfactant as they reflect the chemical structure of the surfactant molecule that dictates the size of the surfactant and its affinity for the interface. To see the shape of the function γ­(*c*; θ), the curve in Figure 1 shows γ­(*c*; θ) with parameters θ fit to the surface tension isotherm data for the OTG.

#### Bayesian Parameter Inference

We adopt a Bayesian approach^25^ to infer the parameter vector θ (of which the CMC is a component) of the surface tension isotherm γ­(*c*; θ) of the surfactant from its surface tension isotherm data D at each iteration of our experimental sequence. Bayesian parameter inference provides two main advantages: (1) quantification of uncertainty about the surface tension isotherm of the surfactant, which we exploit to algorithmically pick the surfactant concentration for the next experiment; and (2) opportunity for injection of prior information/knowledge about the surface tension isotherm of the surfactant, which can potentially reduce the number of experiments needed to estimate the CMC with high credibility.

Following the Bayesian approach to this inverse problem,^33–35^ we treat the parameter vector θ as a random variable Θ and model its probability density function. Though we are primarily interested in the CMC c* of the surfactant, we must estimate the other parameters *a* and *k* of the surfactant jointly with the CMC. We model our uncertainty in γ_0_ as well, despite directly measuring it. A concentrated probability density indicates certainty, while a spread-out density indicates high uncertainty about the parameters. The origins of this uncertainty are (a) the presence of measurement noise in our surface tension measurements and (b) an insufficient amount of surface tension data to fully characterize the surface tension isotherm.

Bayesian parameter inference, which we conduct after each experiment in the sequence, follows three stages. (1) Preceding the sequence of experiments, we construct a *prior density* of Θ, *p*(θ) to express our knowledge and beliefs about the surface tension isotherm of the surfactant before conducting any experiments. (2) Within the experimental sequence and in possession of a surface tension isotherm data set D , we construct a *likelihood function* p(D|θ) that scores each parameter vector θ according to the consistency between (a) the surface tension isotherm model γ­(*c*; θ) with that parameter vector and (b) the surface tension isotherm data D . (3) Finally, we use the likelihood to update the prior distribution *p*(θ) in light of the data D to obtain the *posterior density* of Θ conditioned on the data, using Bayes’s theorem:

3 $$p\left(\theta |D\right)=\frac{p\left(D|\theta \right)p\left(\theta \right)}{\left[p\left(D\right)=\int_{R^{l}}p\left(D|\mathrm{\theta ̂}\right)p\left(\mathrm{\theta ̂}\right)\;d\mathrm{\theta ̂}\right]}$$

The posterior density encapsulates our current state of knowledge (gained from the data D ) and belief about the parameters of the surfactant, including its CMC. We elaborate in more detail below.

##### The Prior Density

Before we initiate the experimental sequence, we write down a prior probability density^25^ of the parameters *p*(θ) to express what we think the parameters of the surface tension isotherm γ­(*c*; θ) of the surfactant could be. The prior is subjective, to an extent, since different researchers may write down different prior densities. However, the prior for a surfactant can be strongly grounded in empirical data, knowledge, and physical constraints—to the extent that researchers could reach a consensus on a reasonable prior. For example, researchers could agree on a reasonable interval in which the CMC of the surfactant belongs by comparing its chemical structure with other surfactants whose CMCs are known. Depending on our level of uncertainty about a parameter, our prior density can range from informative to diffuse. An informative prior concentrates its density around some rough estimate for the parameter, while a diffuse prior is flat and spread-out.

Our prior for the surface tension of pure water γ_0_ is informative:

4 $$\Gamma_{0}∼N\left(\gamma_{0,\mathrm{obs}},\sigma^{2}\right)$$

a Gaussian distribution centered at our measurement of the surface tension of pure water γ_0, obs_ ≈ 0.0718 [N/m], and endowed with a standard deviation σ = 0.001 [N/m], to reflect the degree of noise that we assume contaminates our surface tension measurements.

For the Langmuir parameter *k* and size parameter *a* of the surfactant, we adopt diffuse priors to reflect our high degree of uncertainty:

5 $$K∼U\;\left(0.0\;m^{3}\text{/}\mathrm{mol},\;10000.0\;m^{3}\text{/}\mathrm{mol}\right)$$

6 A∼U(0.001N/m,0.1N/m)

which are uniform distributions granted with a generous range.

Finally is the prior for our cherished parameter, the CMC of the surfactant. Depending on the surfactant, we adopt either a uniform prior:

7 $$C^{*}∼U\;\left(c_{\min}^{*},\;c_{\max}^{*}\right)$$

or a log-uniform prior:

8 $$\log C^{*}∼U\;\left(\log\left(c_{\min}^{*}\right),\;\log\left(c_{\max}^{*}\right)\right)$$

where [*c*_min_^*^, *c*_max_^*^] is the interval in which we think the CMC could lie. We adopt the log-uniform prior when we are uncertain about the order-of-magnitude of the CMC of the surfactant, whereas the uniform prior is more appropriate when we know the order-of-magnitude of the CMC. Arbitrarily, we will ascribe a uniform prior on the CMC of OTG and a log-uniform prior for the CMC of Triton X-100 to explore both scenarios. Note, we impose a diffuse [log-]­uniform prior on the CMC with generous bounds rather than an informative prior to construct a scenario where we possess little prior information about the CMC of the surfactants. Thereby, we give BED a wide degree of freedom so we can probe its ability to guide our experiments. In practice, the construction of a prior density for the CMC would be based on a comparison of the chemical structure of the surfactant at hand with other surfactants whose CMCs are known.

##### The Likelihood Function

Now, at some point within our experimental sequence, we possess surface tension isotherm data D . The likelihood function quantifies the support the data D lend to each parameter vector θ, according to how well the surface tension isotherm model γ­(*c*, θ) with that parameter vector θ matches the data points in D .

We view our experiment that generates a data point (*c*, γ_obs_) as a stochastic process. Despite following the best experimental protocols, experiment-to-experiment variability in the measured surface tension γ_obs_ of a solution with target surfactant concentration *c* is always present due to the random, incorrigible errors associated with all measurements due to, e.g., electrical noise, vibrations, precision of the glassware, etc.

To account for experimental noise in our surface tension measurements, we treat the measured surface tension γ_obs_ of a solution with target surfactant concentration *c* as a realization of a random variable Γ_obs_. Invoking our thermodynamic surface tension isotherm model γ­(*c*; θ), we model the probability density of Γ_obs_ conditioned on the model parameter vector θ as a Gaussian distribution centered at γ­(*c*; θ) and with standard deviation σ = 0.001 [N/m] to reflect the experiment-to-experiment variation in the measured surface tension:

9 $$\Gamma_{\mathrm{obs}}|\theta ,c∼N\;\left(\gamma \left(c;\theta \right),\sigma^{2}\right)$$

We assume each experiment generating a measured surface tension is independent. To justify the level of experimental noise σ = 0.001 N/m, we take the surface tension as the average over three Wilhelmy plate dips, and the standard deviation among these measured surface tensions is always less than 0.001 N/m. More, to a first approximation, the noise is homoskedastic, i.e., independent of surfactant concentration *c*.

The likelihood function, which is the probability density of the data given the parameter vector, follows from eq 9:

10 $$p\left(D|\theta \right)=\overset{N}{\underset{i=1}{\prod }}\frac{1}{\sqrt{2\pi \sigma^{2}}}\;\exp\left[-\frac{1}{2}{\left(\frac{\gamma \left(c_{i};\theta \right)-\gamma_{i,\mathrm{obs}}}{\sigma }\right)}^{2}\right]$$

The factorization is due to independence among experiments. The likelihood function evaluated at a given parameter vector θ is high (low) if the corresponding model surface tension isotherm γ­(*c*; θ) is close (distal from) to the data. Large errors |γ_*i*,obs_ – γ­(*c*_*i*_, θ)| penalize the likelihood, while σ dictates what constitutes a large error. N.b., we omit the possibility of model discrepancy (bias)^36^ between the true underlying surface tension isotherm and our model γ­(*c*; θ_opt_) with parameters θ_opt_ identified from a large, dense, wide-spanning surface tension isotherm data set.

##### The Posterior Density

Finally, combining the prior distribution *p*(θ) and likelihood function p(D|θ) gives the posterior distribution p(θ|D) via Bayes’s theorem in eq 3. The posterior probability density at the parameter vector θ (1) is proportional to the product of the prior and likelihood at that θ, and thus (2) reflects a blend of (a) the knowledge we gained about the parameters from the surface tension isotherm data D and (b) our prior knowledge. As the data set D grows in size and densely covers the appropriate range of surfactant concentrations, the likelihood overrides or “washes away” the prior distribution.

We are particularly interested in the marginal posterior distribution of the CMC of the surfactant, which, for our specific surface tension isotherm model in eq 2, is

$$p\left(c^{*}|D\right)=\int_{R^{3}}p\left(\theta =\left[\gamma_{0},a,k,c^{*}\right]|D\right)\;d\gamma_{0}\;da\;dk$$ 11

This posterior density of the CMC reflects our current state of knowledge and belief about the CMC of the surfactant at this point in the experimental sequence with surface tension isotherm data D .

#### Sampling from the Posterior Density

The evidence term p(D) in the denominator of Bayes’s theorem in eq 3 is just a constant—a normalizing factor for the posterior. Yet, the evidence term is troublesome to compute; the l -dimensional integral generally will not admit a closed-form solution.

So, we resort to Markov chain Monte Carlo (MCMC) sampling to obtain samples (θ_1_, ..., θ_*N* *s*_) from the posterior distribution p(θ|D) . Then, we approximate the posterior of the CMC $p\left(c^{*}|D\right)$ with a kernel density estimator trained on the CMC component of the parameter samples (θ_1_, ..., θ_*Ns*_).

MCMC sampling involves constructing a Markov chain whose (a) state space is the parameter space $R^{l}$ and (b) stationary distribution matches the posterior distribution p(θ|D) . When this Markov chain for *N*_*s*_ steps [after an initial “burn-in” period to reach a state with a high density] is simulated with large *N*_*s*_ values, it gives [autocorrelated] samples from the posterior (θ_1_, ..., θ_*Ns*_). A key advantage of MCMC is that the transition kernel of the MC from state θ to θ′ depends on the ratio of densities $p\left(\theta^{\prime }|D\right)/p\left(\theta |D\right)$ , allowing the intractable evidence term in the denominator of eq 3 to cancel. We use the No-U Turn (NUTS) sampler,^37^ an adaptive variant of the Hamiltonian MCMC sampler that proposes state transitions that follow the geometry of the posterior. NUTS tends to explore the posterior with higher sample efficiency than other (e.g., the random walk Metropolis) MCMC samplers.

Notably, taking the CMC component of the MCMC samples (θ_1_, ..., θ_*N* *s*_) from the posterior p(θ|D) gives samples (*c*_1_^*^, ..., *c*_*N* s_^*^) from the marginal posterior of the CMC $p\left(c^{*}|D\right)$ in eq 11.

MCMC convergence diagnostics^38^ involve running multiple chains in parallel and then (a) comparing within-chain vs between-chain variance, (b) drawing trace plots of the parameters over iterations, and (c) visualizing chain-to-chain consistency of the marginal posteriors.

### Quantifying Uncertainty about the CMC with Information Entropy

We quantify our uncertainty about the CMC when in possession of surface tension isotherm data D using the information [differential] entropy of the marginal posterior density of the CMC, $p\left(c^{*}|D\right)$ :

12 $$S[p\left(c^{*}|D\right)]≔-\int_{R}p\left(c^{*}|D\right)\;\log\left[p\left(c^{*}|D\right)\right]\;dc^{*}$$

The entropy *S*[·] is a functional of the posterior probability density of the CMC that measures its spread over the surfactant concentration line. A high (low) entropy of the posterior density of the CMC reflects a high (low) uncertainty; e.g., the entropy of a Gaussian distribution grows with the logarithm of its standard deviation, and for a given support interval, entropy is maximal for a uniform distribution (complete uncertainty) and minimal for a Dirac delta distribution (complete certainty). In our effort to estimate the CMC of a surfactant with high credibility, we want the entropy of the posterior of the CMC to be small.

#### Approximating the Entropy from Samples

We approximate $S[p\left(c^{*}|D\right)]$ in eq 12 from the posterior samples of the CMC (*c*_1_^*^, ..., *c*_*N* s_^*^). First, we approximate the continuous density $p\left(c^{*}|D\right)$ using a kernel density estimator (Gaussian kernel with Silverman’s rule for the smoothing bandwidth) built on the samples of the CMC. Second, we use adaptive numerical quadrature to integrate the kernel density estimator over the support of the prior distribution of c*.

### Experimental Design Strategy

Finally, we describe our policy that automatically picks the surfactant concentration for the next surface tension measurement in the experimental sequence. We designed our automated decision-making algorithm with the ultimate aim of gaining information about the CMC from the hypothetical data point obtained in the next experiment. Each decision is based on (1) our current belief and knowledge about the surface tension isotherm of the surfactant (including its CMC) encapsulated in the posterior distribution p(θ|D) and (2) anticipation of noise contaminating our future surface tension measurement. We follow ref 20 in explaining the decision-making process in BED.

Hypothetically, consider the next experiment, where we measure the surface tension of a solution with surfactant concentration *c*′, giving a hypothetical new data point (*c*′, γ_obs_^′^). Subsequently, we would augment our current data set D with this new data point and compute the new, updated posterior of the CMC.

13 $$p(c^{*}|D\cup \left\{\left(c^{\prime },\gamma_{\mathrm{obs}}^{\prime }\right)\right\})$$

Our information gain about the CMC from this new data point is the associated reduction in the entropy (i.e., uncertainty) of the posterior of the CMC.

14 $$IG_{c*}\left(c^{\prime },\gamma_{\mathrm{obs}}^{\prime };D\right)≔S[p\left(c^{*}|D\right)]-S[p(c^{*}|D\cup \left\{\left(c^{\prime },\gamma_{\mathrm{obs}}^{\prime }\right)\right\})]$$

We want to pick *c*′ for the next experiment that gives the maximal information gain about the CMC. Unfortunately, though, we do not know what the value of the measured surface tension γ_obs_^′^ in a new hypothetical experiment would be to ascribe a single information gain to each choice of *c*′. However, by sampling a parameter vector θ from our posterior p(θ|D) then generating a measured surface tension γ_obs_^′^ via eq 9, we can predict/simulate/sample outcomes of our hypothetical next experiment with surfactant concentration *c*′. The next measured surface tension Γ_obs_^′^ is a random variable owing to both measurement noise and epistemic uncertainty about the surface tension isotherm of the surfactant. Consequently, our experimental design strategy is to pick the surfactant concentration *c*_opt_^′^ for the next experiment that maximizes the *expected information gain* about the CMC, EIG_c*_:

15 $$\begin{array}{c} c_{\mathrm{opt}}^{\prime }≔\underset{c^{\prime }\in \left[c_{\min}^{*},c_{\max}^{*}\right]}{\mathrm{argmax}}EIG_{c*}\left(c^{\prime }\right)\\ EIG_{c*}\left(c^{\prime }\right)≔E_{p\left(\theta |D\right)p\left(\gamma_{\mathrm{obs}}^{\prime }|\theta ,c^{\prime }\right)}\left[IG_{c*}\left(c^{\prime },\gamma_{\mathrm{obs}}^{\prime };D\right)\right] \end{array}$$

In summary, we view our experimental sequence as a quest for information about the CMC. Accordingly, our adaptive experimental design principle is to choose the surfactant concentration for the next experiment that maximizes the expected information gain about the CMC. Equivalently, since the current entropy of the posterior of the CMC is constant, we minimize the expected entropy of the posterior of the CMC after augmenting our current surface tension isotherm data with the new hypothetical data point. The posterior distribution over surface tension isotherms of the surfactant and our stochastic model of noise corrupting the surface tension measurements are both key to predicting the outcomes of future hypothetical experiments to actually compute the expected information gain about the CMC.

#### Computing the Expected Information Gain

We compute the expected information gain about the CMC, EIG_c*_(*c*′), for a given surfactant concentration *c*′, as follows. First, we compute the entropy of the current posterior of the CMC, $S[p\left(c^{*}|D\right)]$ , the first term in eq 14, which is constant with respect to the expectation E[·] . Second, we repeatedly (1) sample a parameter vector θ from the [precomputed and represented by MCMC samples] posterior density p(θ|D) ; (2) simulate a surface tension measurement γ_obs_^′^ by adding Gaussian noise (std: σ) to the predicted surface tension γ­(*c*′; θ); (3) run the MCMC sampler with the augmented data set to draw samples from the predicted new posterior, $p(\theta |D\cup \left\{\left(c^{\prime },\gamma_{\mathrm{obs}}^{\prime }\right)\right\})$ ; then, from these samples, (4) estimate the entropy of the new predicted posterior of the CMC, $S[p(c^{*}|D\cup \left\{\left(c^{\prime },\gamma_{\mathrm{obs}}^{\prime }\right)\right\})]$ , via (a) constructing a kernel density estimate of $p(c^{*}|D\cup \left\{\left(c^{\prime },\gamma_{\mathrm{obs}}^{\prime }\right)\right\})$ using the MCMC samples from (3) then (b) estimating the value of the integral in eq 12 with this density using adaptive numerical quadrature. Averaging the entropies of the new predicted CMC posteriors over many simulated realizations of surface tension measurements gives the expectation of the second term in eq 14. Notably, this is a rather computationally expensive process.

#### Maximizing the Expected Information Gain

We do an exhaustive grid search for the surfactant concentration *c*_opt_^′^ that maximizes the expected gain of information about the CMC, EIG_c*_(*c*′). The grid points on the surfactant concentration line span the support of the prior of the CMC. Generally, the grid of candidate surfactant concentrations for the next experiment should span a larger range than the bulk of the support of the prior distribution over the CMC. After all, surface tension measurements far from the CMC can still provide a great deal of information about the CMC. For the uniform (log-uniform) prior on the CMC, the grid point spacing is uniform (logarithmic). We increase the resolution of the grid as the search proceeds. A more efficient gradient-free optimization algorithm, e.g., the golden-section search, could reduce the computational cost for decision-making. We adopted the grid search to (i) avoid settling in a local (not global) maximum and (ii) visualize the expected information gain as a function of the surfactant concentration for the next experiment.

#### Factors That Affect the Decision Sequence

Since BED is adaptive, the surfactant concentration it picks for the next experiment depends on the surface tension isotherm data from all previous experiments. Though BED explicitly acknowledges and models the presence of experiment-to-experiment variation in the measured surface tension through eq 9, the decision sequence is affected by noise that contaminates the measured surface tension. The decision sequence also depends on the prior distribution of the surface tension isotherm parameters.

### Posterior-Derived Oracle for a Baseline: A Static, Uniform Design

As a baseline against BED at every iteration, we simulate outcomes of static designs where the surfactant concentrations are [log]­uniformly spaced across the support of the prior distribution over the CMC. As collecting experimental surface tension isotherm data for *N* = 7 uniform experimental designs for baselines would be too time-consuming, we simulate these scenarios by constructing an “oracle” derived from our final posterior over the surface tension isotherm parameters. The oracle generates a simulated measurement of the surface tension γ_obs_ of a solution with surfactant concentration *c* by (1) sampling a parameter vector θ from the final (i.e., built from all data obtained from the BED sequence) posterior p(θ|D) and (2) adding Gaussian-distributed (std: σ) noise to the model prediction γ­(*c*; θ). Since we initialize BED with two manually chosen experiments, the appropriate comparison is BED at iteration *N* versus a static, [log]­uniform design of *N* + 2 experiments with surface tension isotherm data generated by the oracle. At each iteration *N* of BED, we compute the expected entropy of the posterior of the CMC under the simulated size *N* + 2 surface tension isotherm data given by the oracle under a [log]­uniform spacing of the surfactant concentrations. To validate the oracle, we ask the oracle for surface tension isotherm data involving the same surfactant concentrations chosen by BED and compare the entropy of the CMC posterior with that from BED.

### Software

We implement our Bayesian inference routine in the probabilistic programming package Turing.jl^39^ within the Julia programming language. The raw data and Julia code to reproduce our work are openly available on Github at https://github.com/SimonEnsemble/active_learning_cmc.

## Results and Discussion

We now apply Bayesian experimental design (BED) to adaptively design a sequence of experiments aimed at efficiently determining the CMC of two surfactants: (1) OTG and (2) Triton X-100. We allocate a budget of nine experiments for each campaign.

### Case 1: Surfactant OTG (Uniform Prior over the CMC)

At the outset, we invoked a uniform prior distribution for the CMC of the OTG over the interval [0, 30] mol/m^3^. We selected 30 mol/m^3^ as the upper bound, as it is well above the CMC of common nonionic surfactants. We initialized the sequence of experiments with two [manually designed] experiments at the two concentration extremes of the support of the prior over the CMC. The first data point is a direct measurement of the γ_0_ parameter of the surface tension isotherm model; the last data point indicates the surface tension in the flat region of the surface tension isotherm. For the remaining experiments, our BED algorithm picked the surfactant concentrations.

We allowed our BED algorithm to design a sequence of seven more experiments. At each iteration, we, on our computer, (1) used all OTG surface tension isotherm data obtained thus far to compute the posterior distribution over the surface tension isotherm of the OTG and, then, (2) picked the surfactant concentration for the next experiment that maximized the expected information gain about the CMC of the OTG; then, we went into the lab to (3) prepare that surfactant solution and measure its surface tension to obtain another data point on the surface tension isotherm. Summarizing the outcome of the BED sequence, Figure 3A shows the posterior distribution and credible interval of the CMC and Figure 3B shows the acquired data in the experimental sequence and posterior distribution over the surface tension isotherm.

**Figure 3:**
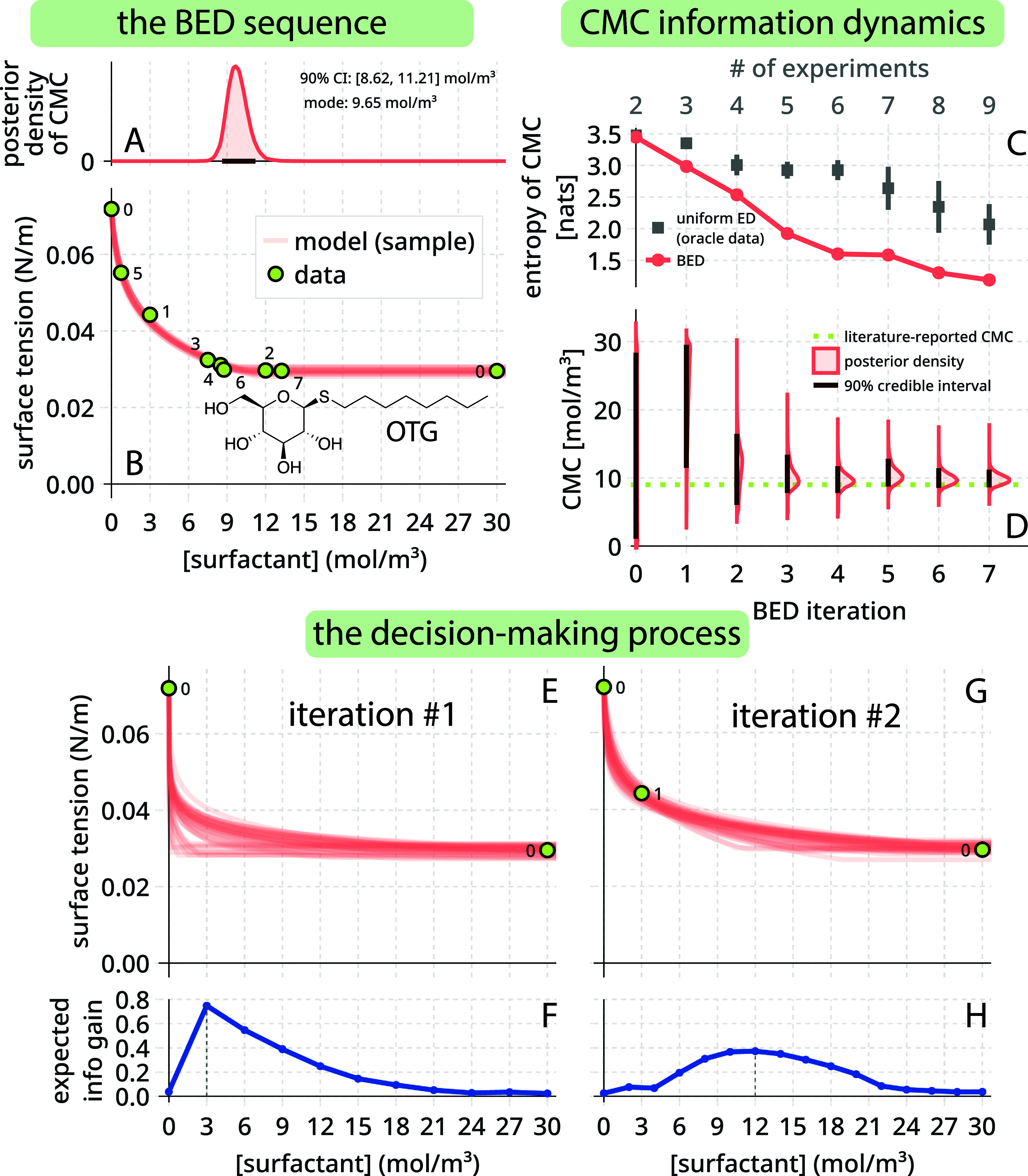
Adaptive, Bayesian design of a sequence of experiments for determining the critical micelle concentration (CMC) of surfactant OTG. (A, B) Summary of the experimental sequence. (A) The posterior distribution of the CMC and its 90% equal-tailed credible interval. (B) Surface tension isotherm data (points) acquired, labeled by the iteration in which they were acquired, and 50 samples of surface tension isotherm models (curves) from the posterior. (C, D) The dynamics of the posterior of the CMC over the BED experimental sequence (C, entropy; D, density and credible interval) and uniform baseline in (C). (E–H) During the first (E, F) and second (G, H) iterations, (E) and (G) show the data and associated posterior over surface tension models and (F) and (H) show the expected information gain about the CMC for each candidate surfactant concentration for the next experiment.

First, we look at the sequence of surfactant concentrations selected by BED in Figure 3B. Each data point is labeled with the iteration in which it was acquired. The two initial experiments that we manually designed belong to iteration zero. BED chose a low surfactant concentration (3.0 mol/m^3^) for the first experiment. Intuitively, this seemed to be a sensible choice. With a new data point outside the flat region, likely the case for a low surfactant concentration, we can estimate the downward trajectory of the surface tension isotherm to predict its intersection with the flat region constrained by the last data point. The second experimental design was a much higher surfactant concentration (12.0 mol/m^3^) near the lower end of the credible interval for the CMC at the time. Intuitively, this seems to be a sensible choice for the next design, as remaining in the flat region would rule out many possible CMCs above this concentration. For the third experiment, BED picked a surfactant concentration between that of the first and second iterations (7.5 mol/m^3^). BED then picked a surfactant concentration (8.5 mol/m^3^) for the fourth experiment between the third and second. While the behavior of BED for the first four iterations resembled a bisection algorithm (viewing the flat and nonflat regions as having opposite signs), the fifth and seventh experiments revisit surface tension intervals that a bisection algorithm would have permanently discarded.

Figure 3B also visualizes the final posterior distribution of the surface tension isotherm (curves) at the end of the experimental sequence, while Figure 3A shows the marginal posterior distribution of the CMC (MCMC convergence diagnostics in Figure S2). The posterior samples of the model surface tension isotherm exhibit little variation. Together, the samples looked like a thick line. Meanwhile, the posterior density over the CMC (the quantity of interest) is highly concentrated at its mode of 9.7 mol/m^3^. Contrasting with the uniform prior density with which we started, the concentrated/peaked posterior density reflects the information we gained about the CMC from the surface tension isotherm data. The equal-tailed 90% credible interval for the CMC is [8.62, 11.21] mol/m^3^, agreeing with reports in the literature (Table 1). The width of the credible interval reflects our degree of remaining uncertainty about the CMC, and it is partly attributable to the prescribed variance σ^2^ of our surface tension measurements. This credible interval has an intuitive interpretation (unlike a confidence interval): given the surface tension isotherm data, our data-generating model, and our prior distribution, we now believe the CMC of the OTG is between 8.62 mol/m^3^ and 11.21 mol/m^3^ with 90% probability. Table 1 displays the final posterior credible intervals for all of the parameters, while Table S1 shows the same parameters determined by using a traditional isotherm fitting procedure.

**Table 1:** 90% Equal-Tailed Posterior Credible Intervals for the Parameters of the Surface Tension Isotherm Model of Each Surfactant Based on the Final Posterior Distribution^a^

surfactant	c* (mol/m^3^)	*k* (m^3^/mol)	*a* (N/m)	*c*_reported_^*^ (mol/m^3^)
OTG	[8.62, 11.21]	[2.5, 6.2]	[0.010, 0.013]	8.7–9.3 ^40–42^
Triton X-100	[0.35, 0.54]	[240, 800]	[0.007, 0.009]	0.25–0.45 ^43,44^

a For comparison, we include a range of literature-reported values of the CMCs.

Note in Figure 3B the sparsity of queries in the flat region of the surface tension isotherm, yet confidence throughout. Both features stem from inductive bias: we constrained the surface tension isotherm model to belong to the thermodynamics-grounded family of functions in eq 2, effectively injecting prior information about the shape of the isotherm. In contrast, a nonparametric model of the surface tension isotherm, such as a Gaussian process regressor, exhibites high uncertainty where data are sparse and would lead to redundant queries in the flat region to resolve the trend therein that the thermodynamic model assumes a priori.

We compare the BED method to a traditional approach to determine the CMC. We collected many data points to densely cover the surfactant concentration line and identified the CMC of the OTG using the intersection of an isotherm in the surface tension reduction region and a flat line fit in the plateau region. See Section S1 and Figure S1A. We found a CMC for the OTG of 9.1 mol/m^3^, which falls within our credible interval.

Next, in Figure 3C,D, we look at the progress of BED from iteration-to-iteration in gaining information about the CMC. Figure 3C shows the reduction in the entropy of the posterior distribution of the CMC of the OTG as the experimental sequence proceeded. Each drop in entropy corresponds with a gain in information about the CMC of OTG. A lower entropy corresponds with a more concentrated/less spread-out posterior distribution of the CMC, which Figure 3D shows directly.

As a baseline, we employed our posterior-derived oracle to simulate the outcomes of static experimental designs (10 runs) where the surfactant concentrations are uniformly spaced over [0, 30] mol/m^3^. See Figure S3 for example synthetic surface tension isotherm data sets. Figure 3C shows that the entropy of the posterior of the CMC reduces much more slowly than by using BED. To obtain the same [small] entropy of the posterior of the CMC given by BED after nine experiments, we would need roughly *twice* the number of experiments if we had adopted a static, uniform design (see Figure S4a).

Finally, Figure 3E–H peers into the decision-making process in the first (E, F) and second (G, H) iterations of the BED sequence. Panels E and G show the data we began with in that iteration and samples from the corresponding posterior over the surface tension isotherm of OTG. Panels F and H show the expected information gain about the CMC for each candidate surfactant concentration for the next experiment. We selected the surfactant concentration for the next experiment that gave the highest expected information gain, 3 mol/m^3^ for iteration #1 and 12 mol/m^3^ for iteration #2. Going from two data points in iteration #1 to three data points in iteration #2, note the reduction in (a) variance in the posterior over the surface tension isotherm of the OTG and (b) expected information gain.

### Case 2: Surfactant Triton X-100 (Log-Uniform Prior over the CMC)

For a second case study, we adopted BED to estimate the CMC of surfactant Triton X-100. We imposed a log-uniform prior distribution for the CMC with support from 0.001 to 10 mol/m^3^. Accordingly, we are assuming that we do not even know the order-of-magnitude of the CMC of Triton X-100. We initialize the experimental sequence with two surface tension measurements of (i) pure water and (ii) a concentrated stock solution of Triton X-100 at 10 mol/m^3^.

Summarizing the outcome of BED for gathering information about the CMC of Triton X-100, Figure 4A shows the final posterior distribution of the CMC and Figure 4B, the sequence of surface tension isotherm data acquired by BED and samples of surface tension isotherms from the posterior distribution. Again, for the first experiment, BED picked a low surfactant concentration. From there, the second, third, fourth, fifth, and sixth experiments marched forward incrementally in surfactant concentration. The seventh experiment walked backward to a lower surfactant concentration. These acquisition dynamics for Triton X-100 differ from the BED for OTG, where we imposed a uniform prior instead of a log-uniform prior, as for Triton X-100. The 90% equal-tailed posterior credible interval for the CMC of Triton X-100 is [0.35, 0.54] mol/m^3^, agreeing with reports in the literature (Table 1). Table 1 displays the final posterior credible intervals for all of the parameters, and Table S1 shows these same parameters determined using a traditional approach. The CMC of Triton X-100 used in these experiments, determined by a traditional method, was found to be 0.46 mol/m^3^ (Figure S1B), falling within our credible interval.

**Figure 4:**
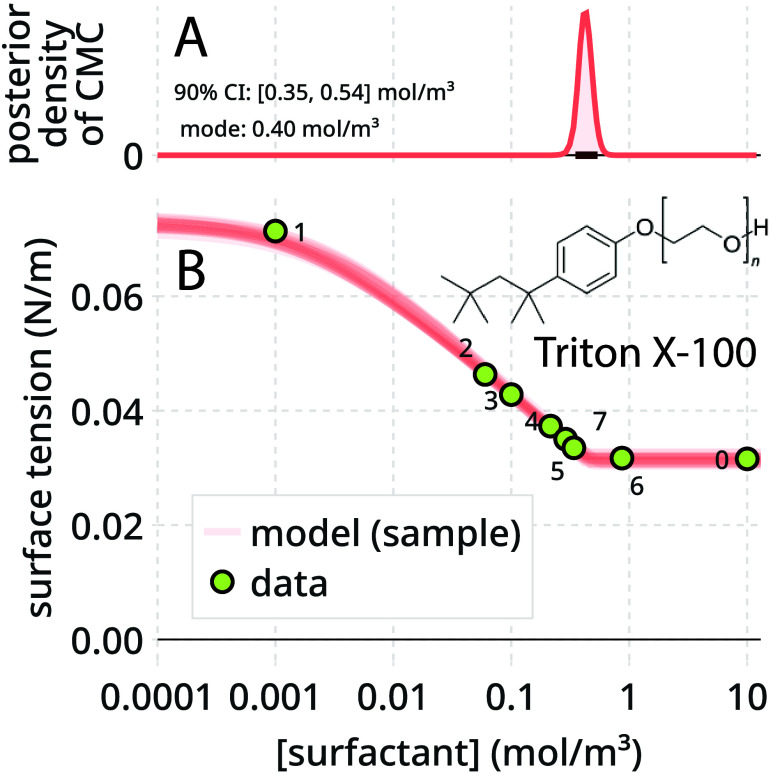
Adaptive, Bayesian design of a sequence of experiments for determining the critical micelle concentration (CMC) of surfactant Triton X-100. (A) The final posterior of the CMC. (B) The sequence of acquired surface tension isotherm data and samples of surface tension isotherm models from the posterior. Note: The data point for pure water was omitted to accommodate the log-scale surfactant concentration.

For the baseline, we employed our posterior-derived oracle to simulate the outcomes of static experimental designs (15 runs), where the surfactant concentrations are log-uniformly spaced from 0.001 to 10 mol/m^3^. Figure S4b shows that, to obtain the same [small] entropy of the posterior of the CMC given by BED after nine experiments, we would need roughly 1.5 times the number of experiments if we had adopted a static, log-uniform design.

### Comparing Cases 1 and 2

The sequence of surfactant concentrations adaptively and automatically chosen by BED were based on the principle of maximizing the expected gain in information about the CMC. For OTG, the decision sequence resembled the behavior of a bisection algorithm for root finding. For Triton X-100, the decision sequence resembled a gradual increment toward the CMC. The decision-making pattern differed between OTG and Triton X-100, likely because we began with a uniform prior for the CMC of OTG, while Triton X-100 began with a log-uniform prior. For each OTG and Triton X-100, while the emergent decision sequence seemed to follow a pattern, it deviated from any simple, static, preprogrammed search algorithm by back-tracking. Arguably, following a search pattern yet back-tracking when deemed necessary matches the behavior of a human experimenter in the lab.

Typically, a human experimenter spends a full day in the laboratory to determine the CMC of a surfactant. This traditionally involves collecting a “full” surface tension isotherm by preparing and measuring the surface tension of ≈20 surfactant solutions spaced out on the concentration line. By adaptively and algorithmically choosing the sequence of surfactant concentrations, BED demonstrates the potential to reduce this human effort in the lab by a factor of 2.

Crucially, the utility of BED for guiding surfactant characterization is predicated on rapid computational decision-making. In our BED implementation, each decision, involving Bayesian inference and expected information gain maximization, took ≈20 min, which is unsatisfactorily time-consuming. Warm-starting the MCMC sampler to reduce the burn-in period, reducing the number of MCMC samples, and running more independent chains in parallel could lower this cost. Additionally, we could dramatically reduce the computational cost of the computational decision-making via adopting (1) variational inference or the Laplace approximation^45^ instead of MCMC sampling to estimate the posterior and expected information gain and (2) an adaptive optimization algorithm^46^ instead of an exhaustive grid search to maximize the expected information gain.^20^

## Conclusions

We outlined and demonstrated an adaptive BED algorithm for picking a sequence of experiments to efficiently determine the CMC of a surfactant in an aqueous solution without prior knowledge of the CMC or even its order of magnitude. Instead of setting the sequence of surfactant concentrations in solutions for surface tension measurements at the beginning of the experimental sequence, our adaptive BED algorithm constituted a feedback loop between (i, experiment) measuring the surface tension of a surfactant solution to obtain a data point; (ii, model) re-inferring the parameters of a thermodynamic surface tension isotherm model of the surfactant; (iii, design) picking the surfactant concentration for the next experiment that provides the largest expected gain in information about the CMC. We demonstrated how BED can reduce the number of experiments needed to determine the CMC of a surfactant with high credibility—with surfactants OTG and Triton X-100 as case studies. Compared with static, [log]­uniform experimental designs (simulated by a posterior-derived oracle), BED reduced the number of experiments required to determine the CMC by a factor of 2 for OTG and a factor of 1.5 for Triton X-100. Our BED algorithm allows for (a) incorporation of prior information about the CMC to increase experiment-efficiency and (b) an elegant experiment-sequence-stopping criterion of estimating the CMC with sufficient credibility.

Broadly, our study makes three contributions. First, our BED algorithm can reduce the time, effort, cost, and chemical waste involved in determining the CMC of surfactants. These experiment-efficiency gains are practically impactful because determining the CMC of a surfactant is a routine task; often, new surfactants are synthesized or discovered, and we seek the CMC of an existing surfactant at different temperatures or in the presence of other solutes in the solution. Second, BED could serve as an automated decision-making engine for a “self-driving” laboratory^47–49^ where robots prepare surfactant solutions, make surface tension measurements, as in the very recent autonomous pendant drop module of Dankloff et al.^27^ and perhaps even synthesize new surfactants around the clock with high reproducibility. Note there have been recently reported automated robots for determining the CMC of surfactants via fluorescence spectroscopy as well.^50,51^ Third, given the simplicity and low cost of these experiments, our study could provide an accessible learning activity for adaptive experimental design in a chemistry laboratory.^31,52^

### Limitations Paired with Extensions and Future Work

As a proof of concept, our study is subject to limitations. Future work is needed to make our BED algorithm more robust and effective across a wide range of surfactants.

First and foremost, our BED algorithm requires a parametric surface tension isotherm model that describes a family of functions that contains the true surface tension isotherm of the surfactant. If, instead, the parametric model is inadequate/insufficiently complex/biased, the decisions about the surfactant concentration for the next experiment will be suboptimal, and the final credible interval for the CMC is unlikely to contain the true value of the CMC.^36^ Deviations from our surface tension isotherm model in eq 2 (the Langmuir-Szyszkowski equation followed by a flat region beyond the CMC) can stem from lateral surfactant–surfactant attractions at the interface;^53^ premicellular aggregation of surfactants in the bulk phase;^54^ under-monolayer adsorption of surfactant;^55^ reorientation or aggregation of surfactant molecules adsorbed at the interface;^56,57^ and surface-active impurities contaminating the solution.^58^ Such a model discrepancy would reveal itself through the emergence of concentration-dependent structure in the residuals (residual: measured minus mean predicted surface tensions) as data are accrued. Future work to handle such systematic deviations from our surface tension isotherm model may include: (1) augmenting the surface tension isotherm model with an empirical model discrepancy term^36,59^ to expand the function space and capture differences between the model surface tension isotherm and the true underlying isotherm or (2) entertaining a portfolio of surface tension isotherm models, imposing a prior distribution over those models, then softly selecting the most appropriate model as the experimental sequence proceeds (Bayesian model selection and averaging). Alternatively, one could adopt a highly flexible, nonparametric model of the surface tension isotherm, like a Gaussian process regressor. However, adopting a statistical regression model largely ignores thermodynamic knowledge about surfactant adsorption and reduces inductive bias, likely trading experiment-efficiency for flexibility in the shape of the surface tension isotherm. To some extent, entertaining the model discrepancy will also incur this sacrifice. Furthermore, introducing model discrepancy could render some parameters nonidentifiable.^60^

Second, we could treat measurement noise, which captures the experiment-to-experiment variation in the measured surface tension of a solution with a given surfactant concentration, differently. We assumed that the variance of the measurement noise was (a) known and (b) the same at every surfactant concentration (homoscedasticity). The variance of the measurement noise input into the BED framework represents our distrust of the surface tension isotherm data. It modulates the width of the credible intervals for the parameters and the strength with which the data override our priors. Alternatively, we could treat (a) uncertainty in the variance of the noise by modeling it as a random variable, imposing a prior distribution for it, and inferring it from the surface tension isotherm data jointly with the isotherm model parameters and/or (b) dependence of the variance of the noise on the surfactant concentration (heteroscedasticity).

Third, our BED algorithm provides a design for a sequence of *single* experiments. We wait for the results of each experiment before designing the next one. However, we may have multiple researchers and instruments to measure the surface tension of the surfactant solutions. Then, we may wish to, at each iteration, pick the *batch* of experiments that maximizes expected information gain.^61^ Then, we assign each available researcher and/or instrument a different surfactant concentration to conduct experiments in parallel for greater time (but not experiment) efficiency.

Fourth, the prior distribution over the CMC could be informed by (i) molecular models and simulations of the surfactant in an aqueous solution to predict its CMC^62–64^ and (ii) supervised machine learning models trained to predict the CMC of surfactants based on their chemical structure.^15,65–69^ To spur machine learning models, the SurfPro database contains experimental properties (including the CMC) of 1624 different surfactants.^70^ Admittedly, it may be uncomfortable to some that the sequence of experiments designed by BED depends on the somewhat subjective prior distribution with which we begin. However, a well-specified informative prior, e.g., for the CMC, a Gaussian centered at the value predicted by a machine learning model with a variance reflecting a well-calibrated confidence, can warm-start the BED feedback loop and reduce the number of experiments needed to obtain a narrow credible interval for the CMC.

Fifth, when applying BED for nonautomated laboratories, human experimenters are more likely to trust algorithmic experimental designs when supported by context-specific explanations—beyond the generic justification of seeking information gain. To provide a counterfactual-style explanation, we can visualize and compare simulated experimental outcomes across different candidate designs. Specifically, using the current posterior over surface tension isotherms and our probabilistic measurement model, we can simulate measuring the surface tension of a new surfactant solution. For different designs (surfactant concentrations), visualizing where this synthetic data point would fall on the surface tension isotherm and how much it would narrow the posterior distribution of CMC may offer insight into the algorithm’s reasoning.

Sixth, in our surface tension isotherm model, we did not include the effect of (a) temperature or (b) the concentration of additional solutes in the water. We may wish to chart the CMC as a function of the temperature and the concentration of various solutes (e.g., ions) in the solution. As this expands the experimental design space, BED could be very useful for this setting.

Finally, to routinely apply BED for efficient surfactant characterization, we must explore strategies to reduce the computational cost of decision-making via code optimization and/or adopting cheaper, approximate methods—such as variational approaches—to estimate the posterior distribution and expected information gain.^20^

## Supplementary Material



## Notes

Surface tension isotherm data densely covering the full concentration range for OTG and Triton X-100, model fit to find the CMC (traditional approach), and tabulated fit parameters; MCMC convergence diagnostics; Realizations of surface tension isotherm data generated by the posterior-derived oracle under a [log]-uniform design for OTG and Triton X-100; Entropy dynamics of the CMC under BED compared with the [log]-uniform design using synthetic data from the posterior-derived oracle (PDF)
