# Supplementary material for: Adaptive, Bayesian Experimental Design to Efficiently Determine the Critical Micelle Concentration of a Surfactant
Source: Langmuir. 2026 Apr 7;42(15):10325–36. doi: 10.1021/acs.langmuir.5c06514 (PMC13104181; doi:10.1021/acs.langmuir.5c06514)
Supplement: Supplementary file 1 [file la5c06514_si_001.pdf]

Supporting Information: Adaptive, Bayesian experimental design to efficiently determine the critical micelle concentration of a surfactant

Makaila Hammond,[†] Qia Ke,[‡] Aryan Deshwal,[¶] Cory M. Simon,[*,‡] and Alison Bain[*,†]

†*Department of Chemistry, Oregon State University, Corvallis, OR. USA.*

‡*School of Chemical, Biological, and Environmental Engineering, Oregon State University, Corvallis, OR. USA.*

¶*Department of Computer Science and Engineering, University of Minnesota, Minneapolis, MN. USA.*

E-mail: cory.simon@oregonstate.edu; alison.bain@oregonstate.edu

Contents

List of Figures **S2**

List of Tables **S2**

S1 Traditional approach to identify the critical micelle concentration **S3**

S2 Trace plots to assess convergence of MCMC **S4**

S3 [Log]-uniform design as a baseline **S5**

List of Figures

S1 Traditional model fits to identify the CMC S3
S2 MCMC convergence diagnostics S4
S3 Surface tension isotherm data sampled from the oracle S5
S4 Static, uniform design baselines S6

List of Tables

S1 Model parameters identified from traditional fitting S3

S1 Traditional approach to identify the critical micelle concentration

A complete set of surface tension measurements was taken for each surfactant (Fig. SS1). The region where the surface tension is decreasing is fit with the Szyzskowski isotherm, where $\gamma_0 = 0.072$ N/m, and the region where the surface tension plateaus is fit to a straight line. The CMC is determined from the intersection of these two lines.

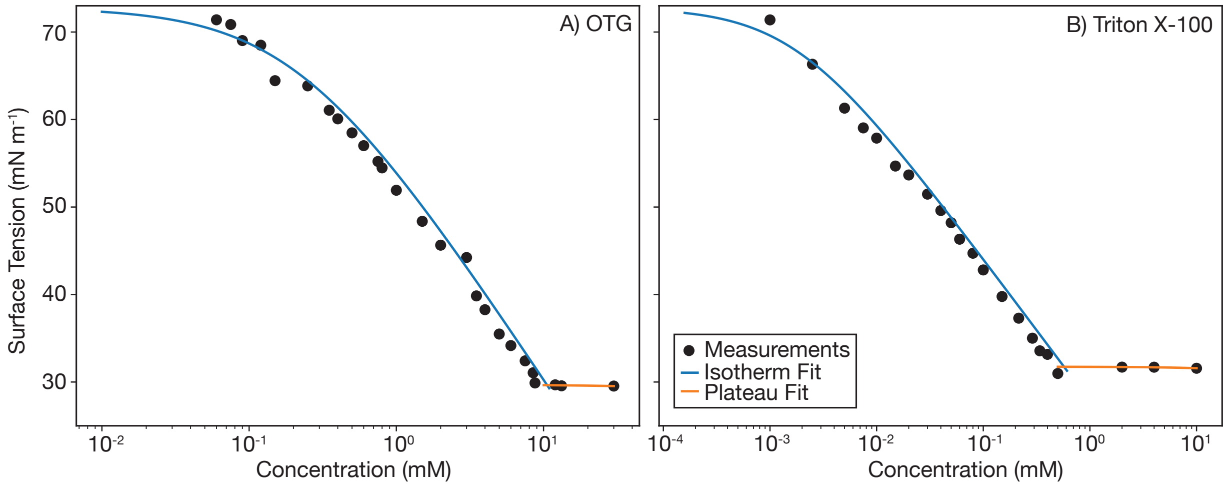

Figure S1: Szyzskowski isotherm and plateau surface tension fits of A) octyl-β-D-thioglucopyranoside (OTG) and B) Triton X-100 solutions used to determine the critical micelle concentration (CMC) using a traditional approach.

Table S1: Fitting parameters and CMC (c^*) for aqueous surfactant found by a traditional approach.

surfactant	c^* (mol/m^3)	k (m^3/mol)	a (N/m)
OTG	9.102 ± 0.002	4.5 ± 0.4	0.0111 ± 0.0004
Triton-X-100	0.46416 ± 0.00005	572 ± 55	0.0071 ± 0.0002

S2 Trace plots to assess convergence of MCMC

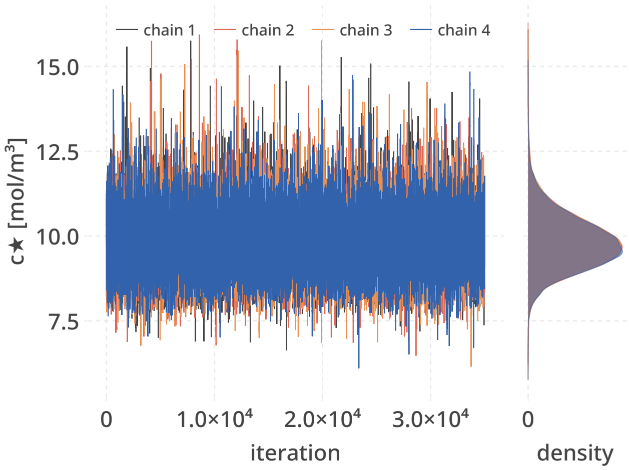

(a) OTG

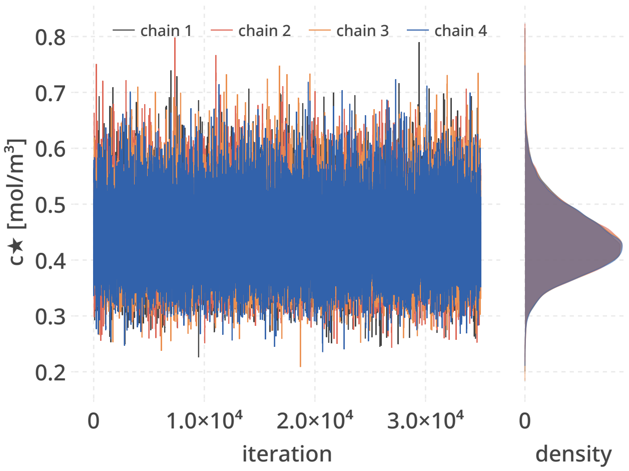

(b) Triton X-100

Figure S2: MCMC convergence diagnostics for the posterior distribution for (a) Triton X-100 and (b) OTG using all nine surface tension isotherm data points collected from BED. The left panel contains a trace plot with the CMC over the course of MCMC sampling for four independent MCs. The sampler thoroughly explores this interval of the CMC and revisits the same sub-interval many times, suggesting convergence. The right panel shows the distribution of the MCMC samples of the CMC for the four chains. The distributions of the MCMC samples of the CMC for the four independent chains agree, also suggesting convergence. (The first 1000 samples discarded for burn-in are not shown.)

S3 [Log]-uniform design as a baseline

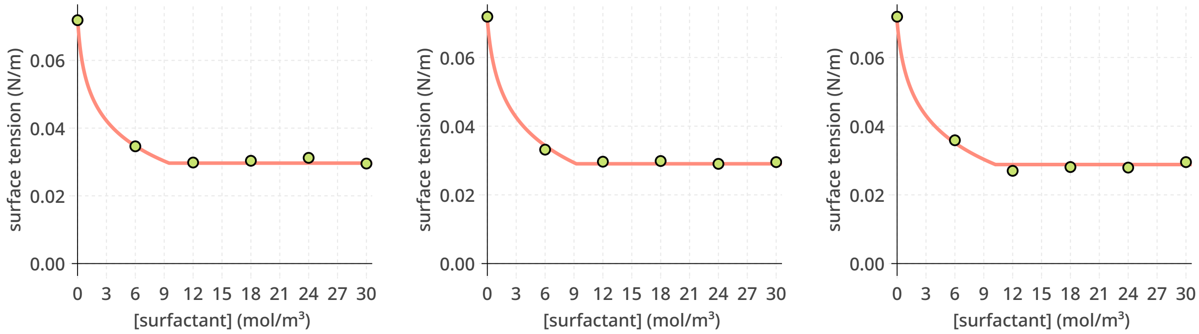

(a) OTG

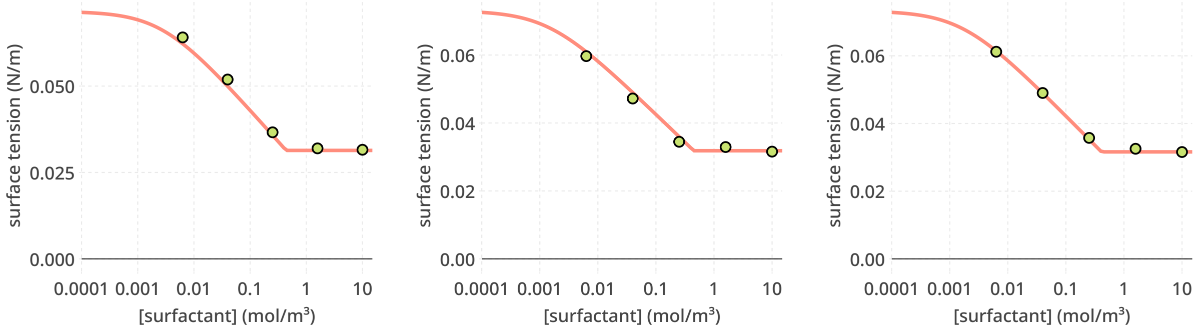

(b) Triton X-100

Figure S3: For illustration, we show three realizations of simulated surface tension isotherm data (points) for (a) OTG and (b) Triton X-100 generated by our posterior-derived oracle, under (a) uniform and (b) log-uniform designs with a budget of six experiments to construct a baseline against BED at iteration four. (The $c = 0$ data with pure water is omitted in (b) to accommodate the log-scale on the concentration axis.) The curve represents the mean of the posterior surface tension isotherm at the end of our BED.

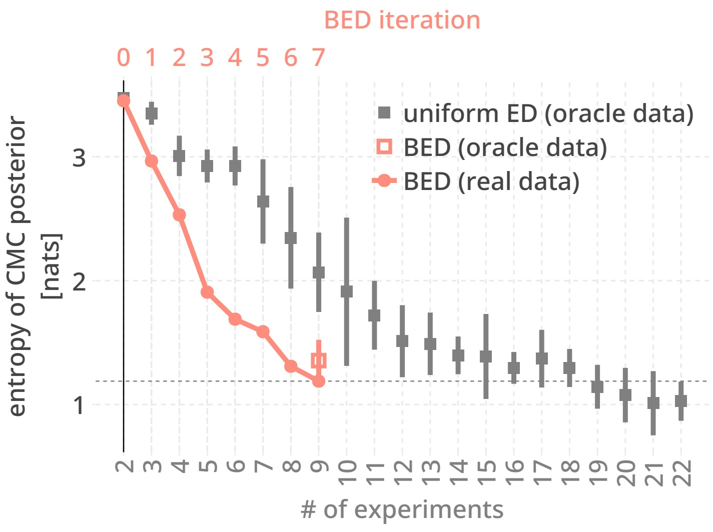

(a) OTG

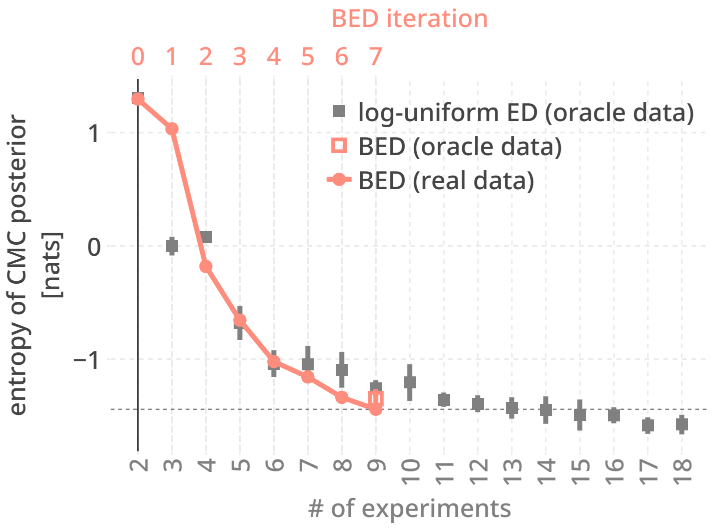

(b) Triton X-100

Figure S4: The dynamics of the information about the CMC of (a) OTG and (b) Triton X-100 over the course of BED (solid orange circles) compared with the [log]uniform design using simulated surface tension isotherm data generated by our posterior-derived oracle (solid gray squares). For the oracle-based data, we show average and standard deviation over (a) 10 and (b) 15 runs. The orange curve and filled circles show the entropy of the posterior of the CMC as a function of iterations for BED, using our real surface tension isotherm data. For a baseline, the solid gray points show the entropy of the CMC (i) according to a (a) uniform or (b) log-uniform design and (ii) based on data generated from the oracle. To validate the posterior-derived oracle, we compute the entropy of the CMC obtained from oracle-generated surface tension isotherm data pertaining to the same surfactant concentrations as in the BED sequence. This is the orange hollow square point.
